# Azithromycin promotes alternatively activated macrophage phenotype in systematic lupus erythematosus via PI3K/Akt signaling pathway

**DOI:** 10.1038/s41419-018-1097-5

**Published:** 2018-10-22

**Authors:** Jie Wang, Lin Xie, Shangshang Wang, Jinran Lin, Jun Liang, Jinhua Xu

**Affiliations:** 1 Department of Dermatology, Huashan Hospital, Fudan University, Shanghai, China; 2 Shanghai Institute of Dermatology, Shanghai, China

## Abstract

Alternatively activated macrophages have been reported to be helpful to alleviate systematic lupus erythematosus (SLE), and azithromycin could serve as an immunomodulator by promoting alternatively activated macrophage phenotype. However, the effect of azithromycin in SLE and the involved mechanism remain undetermined. The aim of this study is to characterize azithromycin and the underlying mechanism contributing to SLE therapy. First, we compared monocytes from SLE patients and matched healthy donors, and found monocytes from SLE patients exhibited more CD14^+^CD86^+^ cells, impaired phagocytic activity, and elevated interleukin (IL)-1β, IL-6, and tumor necrosis factor (TNF)-α (the classical activated phenotype), which could be blocked by azithromycin. On the contrary, there were fewer CD14^+^CD163^+^ cells in SLE patients, accompanied by decreased arginase (Arg)-1 and found in inflammatory zone (Fizz)-1 (the alternatively activated phenotype). And IL-10, the crucial immune regulatory factor secreted by alternatively activated monocytes/macrophages, also showed a decreased trend in SLE patients. In addition, all these markers were up-regulated after azithromycin treatment. Next, we used activated lymphocyte-derived-DNA to imitate SLE macrophages in vitro to investigate the possible mechanism involved. Azithromycin showed the same effect in imitated SLE macrophages, with distinct Akt phosphorylation at 30 min and 12 h. After inhibiting Akt phosphorylation by LY294002, the down-regulation of CD80, IL-1β, IL-6, and TNF-α caused by azithromycin raised again, meanwhile, the up-regulation of CD206, Arg-1, Fizz-1, and IL-10 due to azithromycin was abolished. Additionally, insulin-like growth factor 1 (IGF-1), the specific agonist of Akt, played a similar role to azithromycin in imitated SLE macrophages. Taken together, our data indicated a novel role of azithromycin in alleviating SLE by promoting alternatively activated macrophage phenotype, and the PI3K/Akt signaling pathway was involved. Our findings provide a rationale for further investigation of novel therapeutic strategy for SLE patients.

## Introduction

Systematic lupus erythematosus (SLE) is a multisystem autoimmune disorder which typically affects women of childbearing age. Although the pathogenesis of SLE is not completely clear, one of the crucial concepts generally accepted is the increased apoptosis and/or impaired clearance of apoptotic cells^1,2^. The deposition of excess apoptosis formulates aberrant activation of autoimmunity cells, massive autoantibodies, abnormal cytokines, and ultimately, organ damage, which in turn results in more autoimmune responses^3^.

Macrophages, the innate immune cells, are known for the powerful phagocytosis and high plasticity. In response to bacterial moieties such as lipopolysaccharide (LPS) and T helper type 1 (Th1) cytokine interferon-γ (IFN-γ), macrophages undergo classical activation (M1), characterized by up-regulation of CD80/CD86, secrete substantial pro-inflammatory cytokines such as interleukin (IL)-1, IL-6, and tumor necrosis factor-α (TNF-α)^4,5^. On the contrary, exposure to T helper type 2 (Th2) cytokines such as IL-4 and IL-13 leads to alternative activation (M2), distinguished by up-regulation of scavenger receptor (CD163) and mannose receptor (CD206), increased arginase-1 (Arg-1), found in inflammatory zone (Fizz-1), chitinase-like 3 (Ym-1), IL-10, and more mature phagocytosis^6–8^. In fact, the typical M1 and M2 induced by traditional stimuli (LPS/IFN-γ, IL-4/IL-10/IL-13) are extremes of a spectrum in a galaxy of phenotypic and functional states^9^. Not only the traditional stimuli, but also the local environmental factors could shape macrophage properties ranging from M1 to M2^9–11^. Variations on the theme of M1 or M2 polarization have been also found in vivo, for instance, in the embryo and the placenta, during cancer and obesity, as well as SLE^11–14^. Compared with the lupus-resistant mice, MRL-Fas^lpr^ mice fail to shift the macrophage phenotype from the “destroy” (M1) to the “heal” (M2), the persistent inflammatory infiltration finally triggers lupus nephritis^15^. Additionally, after macrophage depletion and selectively injection of M2, lupus relives magically^16^. The SLE patients who received mesenchymal stem cell transplantation show M2 polarity recovery and increased phagocytosis, accompanied with remission^17,18^. All suggest a potential target for SLE treatment by M2 modulation.

How to obtain the modulation of M2? In recent decade, azithromycin (AZM) was reported to shift macrophage from M1 to M2 phenotype both in vitro and in vivo. In addition to its antibiotic properties, azithromycin could improve M2 activation and phagocytosis in macrophage cell lines, human monocytes, alveolar macrophages, ischemic stroke mice, cord injury mice, and chronic obstructive pulmonary disease (COPD) patients, as well as cystic fibrosis mice and patients^19–25^. Despite the reported immunomodulatory function, the potential therapeutic effects in SLE have not been determined. Moreover, azithromycin showed a strong anti-inflammatory effect comparable to corticosteroids and chloroquine, the major drugs for SLE treatment^20^. Therefore, we hypothesize that azithromycin could improve M2 activation in lupus, inhibit inflammatory factors, and increase phagocytosis, which are beneficial to alleviate SLE.

In this study, activated lymphocyte-derived-DNA (ALD-DNA) was used to imitate lupus in vitro as previously described^10^. We compared the monocytes from SLE patients and matched healthy donors, and detect the role of azithromycin in SLE macrophages. The aim of this study is to investigate the role of azithromycin in SLE and its underlying mechanism.

## Results

### Monocytes from SLE patients showed impaired phagocytic activity and increased pro-inflammatory activity compared to matched healthy controls

**Fig. 1:**
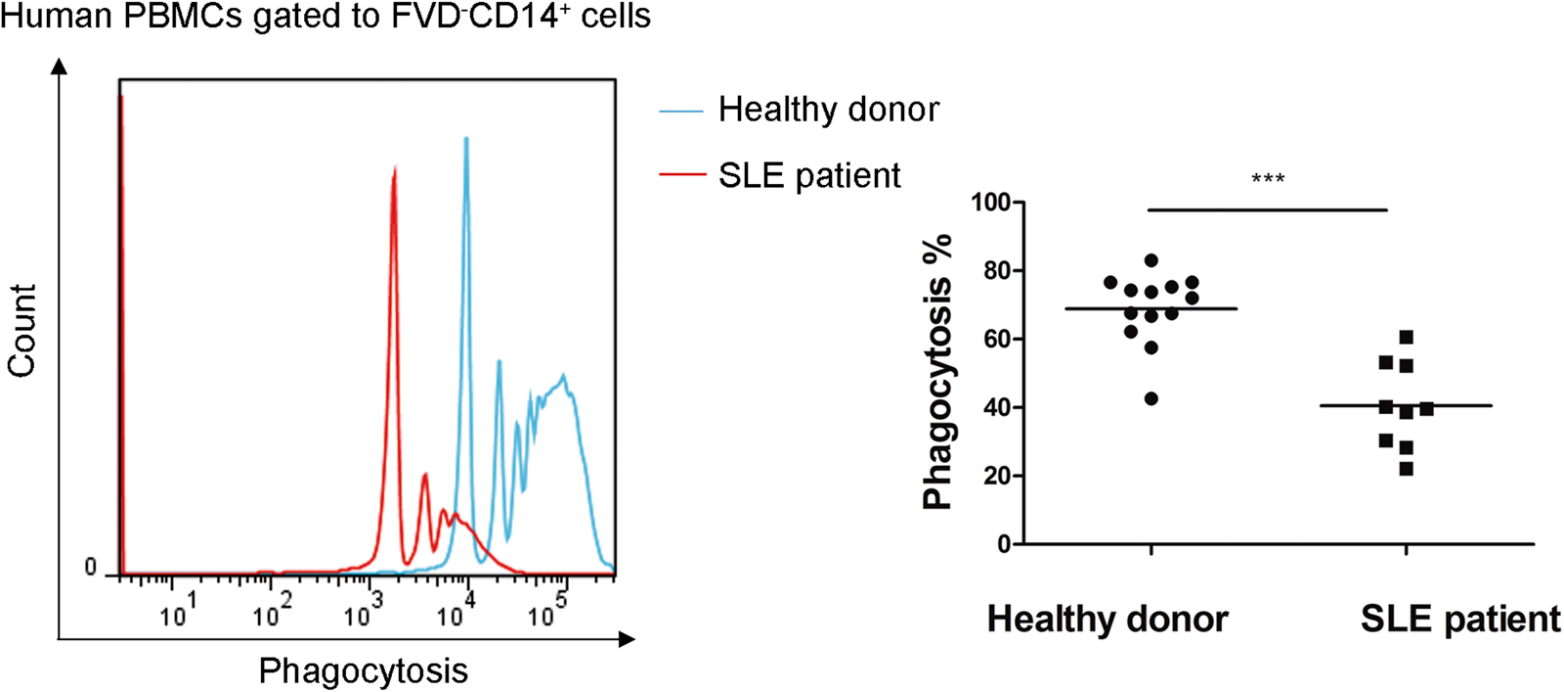
Impaired phagocytic activity of monocytes in SLE patients. Compared with healthy donors (*N* = 13), monocytes from SLE patients (*N* = 9) exhibited impaired phagocytic activity. The blue line indicated healthy donors, and the red one indicated the SLE patients. ***p < 0.001

**Fig. 2:**
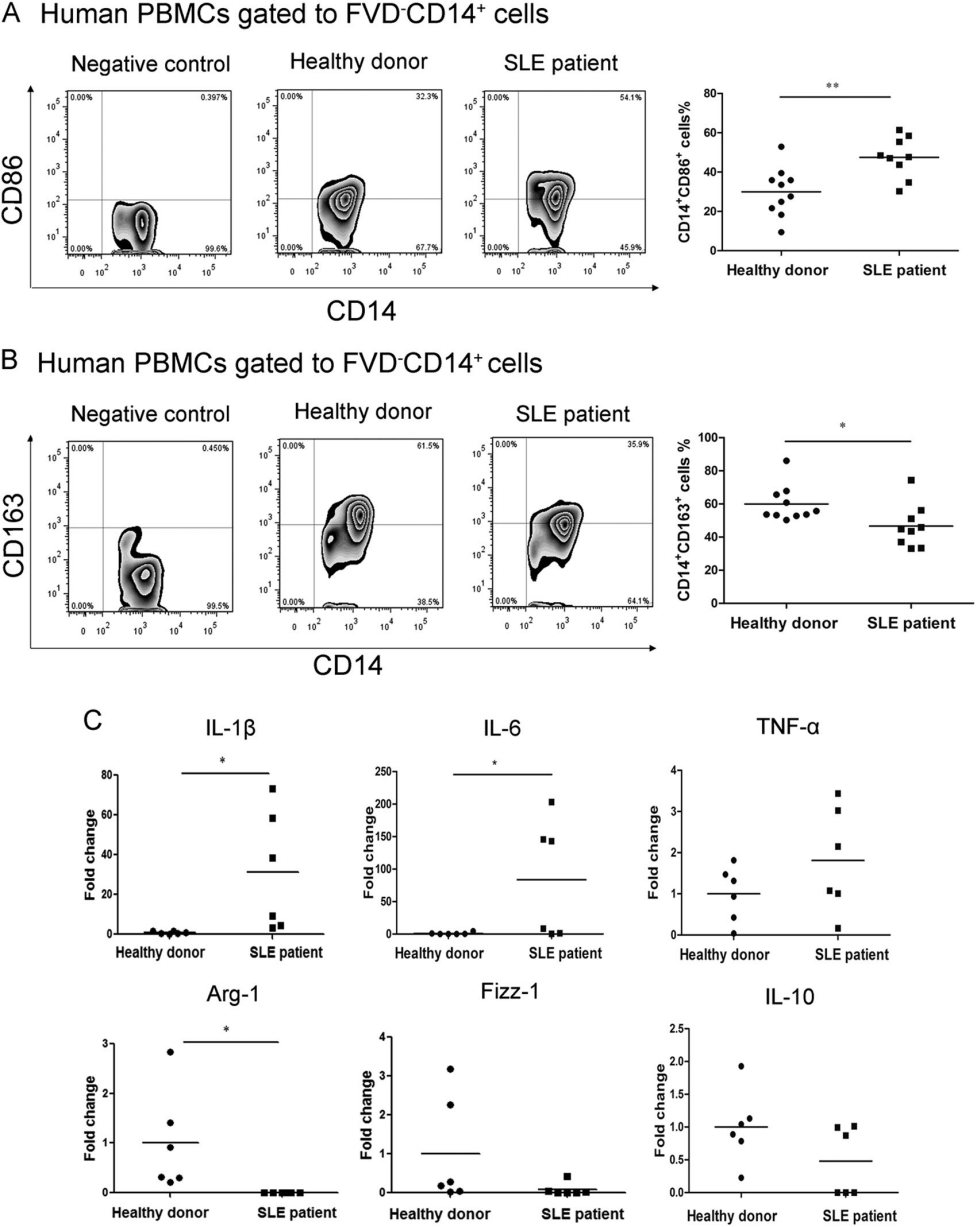
Monocytes from SLE patients displayed a destroyer (M1-like) role. **a** CD14^+^CD86^+^ cells were extremely increased in SLE patients (*N* = 9). **b** CD14^+^CD163^+^ cells were significantly reduced in SLE patients (*N* = 9). **c** The mRNA levels of IL-1β, IL-6, and TNF-α were increased while the mRNA levels of Arg-1, Fizz-1, and IL-10 exhibited a lower trend in SLE patients (*N* = 6). *p < 0.05, **p < 0.01

We compared the phagocytic activity between monocytes from SLE patients and matched healthy controls, and found an extremely lower phagocytic activity in the patients’ monocytes (40.58 ± 12.73% vs. 68.91 ± 10.39%, *p* = 0.000) (Fig. 1). To define the phenotype of monocytes, we used CD14^+^CD86^+^ as the destroyer (M1-like), and CD14^+^CD163^+^ as the repairer (M2-like). Flow cytometry showed increased CD14^+^CD86^+^ monocytes (47.50 ± 10.30% vs. 29.96 ± 12.29%, *p* = 0.004) (Fig. 2a) and decreased CD14^+^CD163^+^ monocytes (46.61 ± 12.98% vs. 59.94 ± 10.86%, *p* = 0.026) (Fig. 2b) in SLE patients. To further examine the function of monocytes, we detected the mRNA levels of IL-1β, IL-6, TNF-α, Arg-1, Fizz-1, and IL-10. Both IL-1β and IL-6 increased significantly in SLE patients (31.26 ± 30.20 fold change and 83.71 ± 90.61 fold change, *p* = 0.034, *p* = 0.049, respectively). The mRNA level of TNF-α showed an increased trend in SLE patients, but was not statistically significant (1.81 ± 1.27 fold change, *p* = 0.197). However, the expression of Arg-1 was undetectable in SLE patients (*p* = 0.036), and a trend of lower mRNA levels of Fizz-1 (0.083 ± 0.17 fold change, *p* = 0.13) and IL-10 (0.48 ± 0.53 fold change, *p* = 0.13) were observed in SLE patients (Fig. 2c).

### Azithromycin increased phagocytic activity and induced M2 properties recovery in human SLE macrophages

**Fig. 3:**
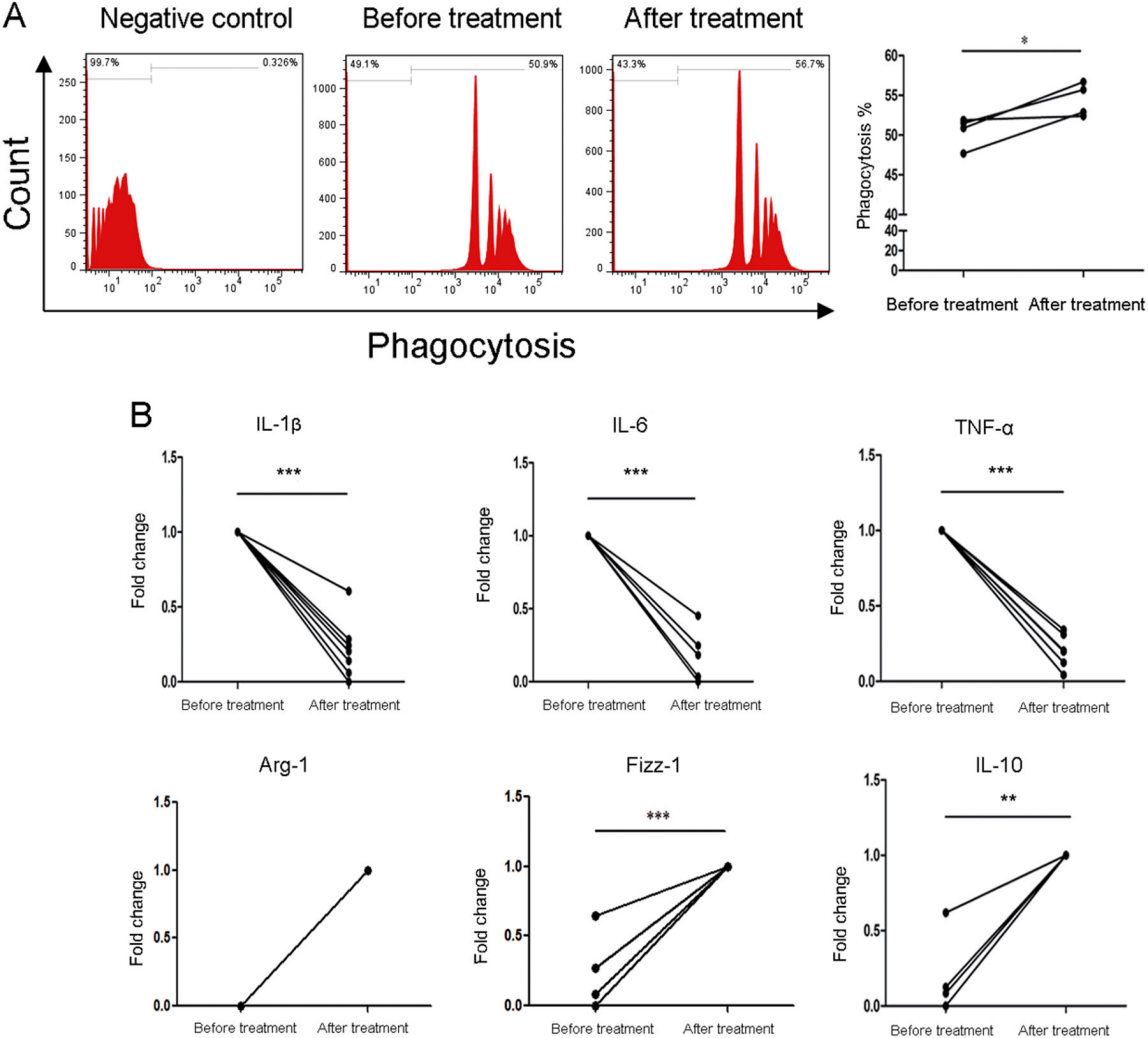
Azithromycin promoted phagocytic activity and M2 phenotype in human SLE macrophages in vitro. **a** Azithromycin improved the phagocytic activity in SLE macrophages (*N* = 4). **b** Azithromycin significantly depressed the mRNA levels of IL-1β (*N* = 7), IL-6 (*N* = 7), and TNF-α (*N* = 6) in SLE macrophages, on the contrary, the mRNA levels of Arg-1 (*N* = 6), Fizz-1 (*N* = 6), and IL-10 (*N* = 5) extremely increased after azithromycin treatment. *p < 0.05, **p < 0.01, ***p < 0.001

After differentiating the SLE monocytes into SLE macrophages with macrophage colony-stimulating factor (M-CSF), cells were treated with azithromycin for another 48 h. The phagocytic activity was increased after azithromycin treatment (54.43 ± 2.10% vs. 50.50 ± 1.91%, *p* = 0.046) (Fig. 3a). Moreover, azithromycin extremely decreased the mRNA levels of IL-1β, IL-6, and TNF-α in SLE macrophages (0.22 ± 0.20 fold change, *p* = 0.000, 0.13 ± 0.17 fold change, *p* = 0.000, 0.20 ± 0.11 fold change, *p* = 0.000), while the mRNA levels of Arg-1, Fizz-1 (*p* = 0.0005), and IL-10 (*p* = 0.002) significantly increased after azithromycin treatment (Fig. 3b).

### Azithromycin promoted M2 phenotype in ALD-DNA imitated SLE macrophages

**Fig. 4:**
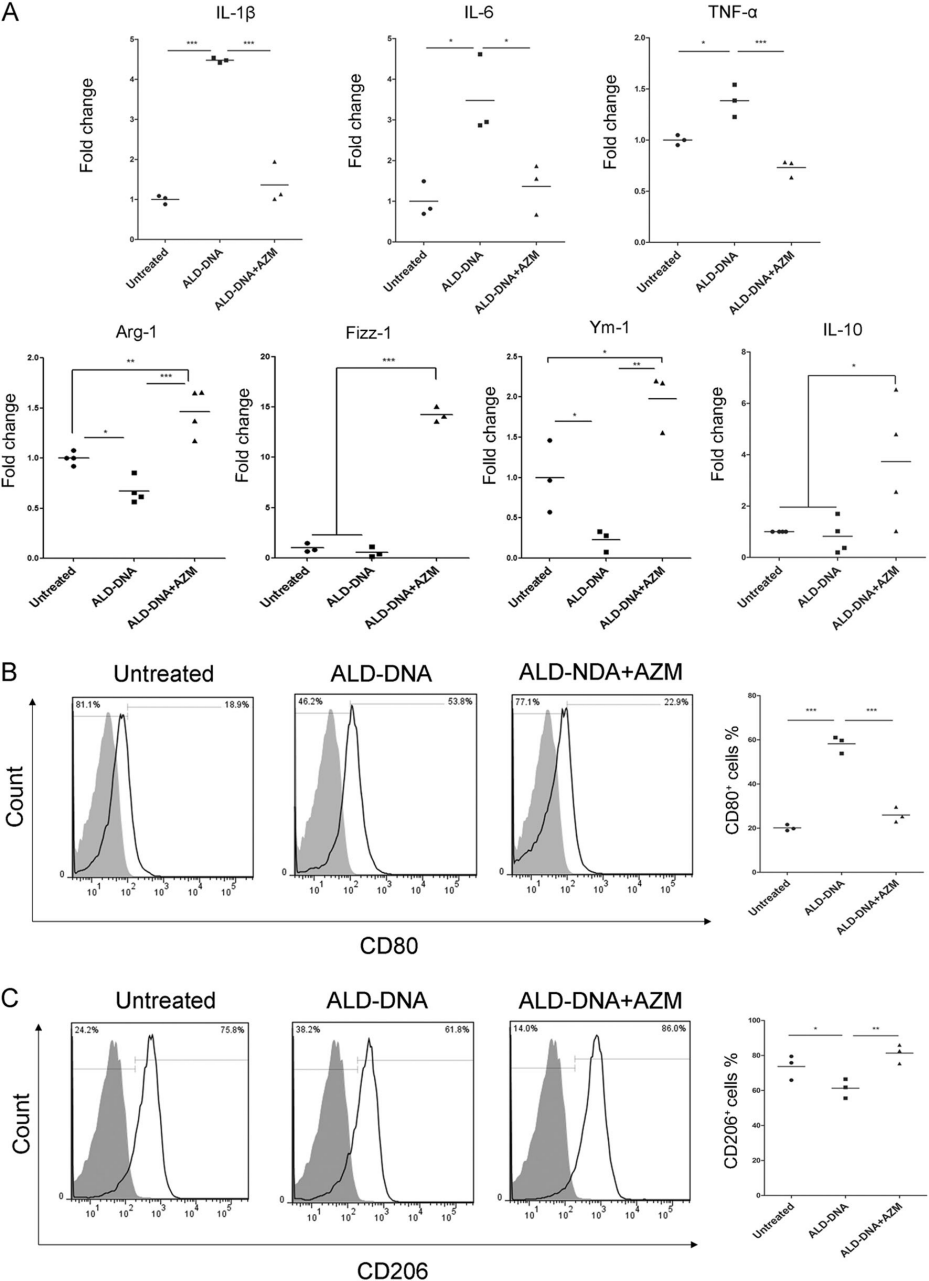
Azithromycin improved M2 recovery in imitated SLE macrophages in vitro. **a** Azithromycin suppressed the increased mRNA levels of IL-1β, IL-6, and TNF-α in ALD-DNA induced cells, however, expressions of Arg-1, Fizz-1, Ym-1, and IL-10 were up-regulated after azithromycin treatment. **b**, **c** CD80 and CD206 expressions were determined by flow cytometer. A representative experiment out of three repeats with similar results is shown. The frequencies of CD80^+^ cells (**b**) or CD206^+^ cells (**c**) are shown in each histogram. Solid black line indicated CD80 or CD206 staining, and filled grey histogram showed negative controls. *N* = 3–4, *p < 0.05, **p < 0.01, ***p < 0.001

To imitate SLE, ALD-DNA was used to induce SLE macrophages in vitro as described previously^10^. More than 98% cells showed apoptosis after stimulation of concanavalin A, and the extracted DNA (the so-called ALD-DNA) exhibited a typical “apoptotic ladder” in the gel (Fig. S1). We found ALD-DNA significantly increased the mRNA levels of IL-1β, IL-6, and TNF-α from the concentration of 10 μg/ml, the fold changes were 4.09 ± 0.85, 3.87 ± 0.97, 1.38 ± 0.14, respectively (*p* = 0.000, 0.001, 0.014, respectively), meanwhile, expressions of Arg-1, Fizz-1, and IL-10 decreased as observed in SLE patients, as well as Ym-1 (Fig. S2). Then, we used azithromycin to treat the imitated SLE macrophages, the expressions of IL-1β (1.36 ± 0.51 fold change vs. 4.48 ± 0.06 fold change, *p* = 0.000), IL-6 (1.36 ± 0.62 fold change vs. 3.48 ± 0.98 fold change, *p* = 0.011) and TNF-α (0.7315 ± 0.08 fold change vs. 1.39 ± 0.16 fold change, *p* = 0.000) reduced extremely after azithromycin treatment, however, the expressions of Arg-1 (1.46 ± 0.23 fold change vs. 0.67 ± 0.13 fold change, *p* = 0.000), Fizz-1 (14.23 ± 0.73 fold change vs. 0.58 ± 0.51 fold change, *p* = 0.000), Ym-1 (1.98 ± 0.36 fold change vs. 0.23 ± 0.14 fold change, *p* = 0.001), and IL-10 (3.73 ± 2.43 fold change vs. 0.82 ± 0.69 fold change, *p* = 0.02) magically increased (Fig. 4a). Meanwhile, as the M1 marker, CD80 was up-regulated in ALD-DNA imitated SLE macrophages (58.17 ± 3.84% vs. 20.10 ± 1.38%, *p* = 0.000), but depressed after azithromycin treatment (25.9 ± 3.40% vs. 58.17 ± 3.84%, *p* = 0.000) (Fig. 4b). On the contrary, the M2 marker, CD206 was declined in ALD-DNA induced macrophages (61.30 ± 5.47% vs. 73.73 ± 7.03%, *p* = 0.045), but raised after azithromycin treatment (81.33 ± 3.12% vs.61.30 ± 5.47%, *p* = 0.007) (Fig. 4c).

### PI3K/Akt signaling pathway was involved in azithromycin-induced M2 activation in imitated SLE macrophages

**Fig. 5:**
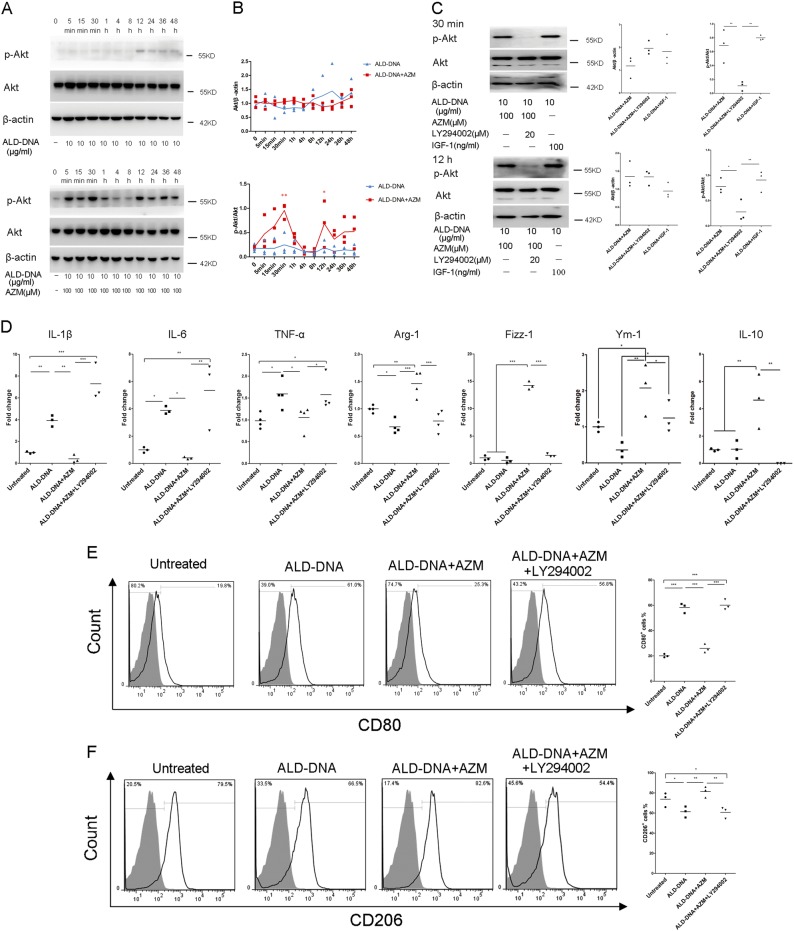
LY294002 blocked the therapeutic effect of azithromycin in imitated SLE macrophages. **a** Raw 264.7 cells were stimulated with ALD-DNA or ALD-DNA+AZM for 48 h. Total Akt and phosphorylated Akt molecules were detected by western blot analysis. One of three experiments with similar results is shown. **b** The ratio of the band density of Akt molecule to β-actin molecule exhibited no significant difference at each time point within or between the two groups. However, the ratio of the band density of p-Akt molecule to Akt molecule was strongly higher in ALD-DNA combined azithromycin-treated cells at 30 min and 12 h, compared with all the other time points and group. The blue one represents ALD-DNA-treated cells and the red one represents ALD-DNA combined azithromycin-treated cells. **c** ALD-DNA-induced cells were treated with LY294002 or IGF-1 in the presence or absence of azithromycin for 30 min or 12 h. Total Akt and phosphorylated Akt molecules were detected by western blot analysis. One of three experiments with similar results is shown. While LY294002 blocked the ratio of p-Akt/Akt at both the 30 min and the 12 h in ALD-DNA combined azithromycin-treated cells, IGF-1 increased the ratio of p-Akt/Akt at the same time point in ALD-DNA-induced cells. **d** LY294002 abolished the azithromycin-induced suppression of IL-1β, IL-6, and TNF-α, as well as the production of Arg-1, Fizz-1, Ym-1, and IL-10. The frequencies of CD80^+^ cells (**e**) or CD206^+^ cells (**f**) are shown in each histogram. Solid black line indicated CD80 or CD206 staining, and filled grey histogram showed negative controls. *N* = 3–4. *p < 0.05, **p < 0.01, compared with the ALD-DNA+AZM group at the initial time. *p < 0.05, **p < 0.01, ***p < 0.001

Last, we investigated the mechanism of azithromycin-mediated M2 activation. We focused on the PI3K/Akt signaling pathway because this pathway is shown to be involved in M2 activation in many cases^26–28^. We found stimulation of azithromycin on imitated SLE macrophages led to a significant increase in Akt phosphorylation at the periods of 5–30 min and 12–48 h, which peaked at 30 min and 12 h (Fig. 5a, b). However, as a control group, the imitated SLE macrophages did not show any Akt phosphorylation during the whole period (Fig. 5a, b). To determine whether PI3K/Akt signaling is involved in azithromycin-induced M2 activation, we stimulated azithromycin-treated cells in the presence of LY294002, a selective inhibitor of PI3K, which is a signaling molecule just upstream of Akt. LY294002 definitely abolished the azithromycin-induced Akt phosphorylation at both the 30 min and the 12 h (Fig. 5c). Consequently, compared with ALD-DNA combined azithromycin-treated cells, the addition of LY294002 increased the mRNA levels of IL-1β, IL-6, and TNF-α (7.30 ± 1.68 fold change vs. 0.41 ± 0.37 fold change, *p* = 0.000, 5.34 ± 2.39 fold change vs. 0.41 ± 0.08 fold change, *p* = 0.002, 1.58 ± 0.38 fold change vs. 1.06 ± 0.28 fold change, *p* = 0.025,) (Fig. 5d) and improved CD80 expression (60.07 ± 4.12% vs. 25.93 ± 3.39%, *p* = 0.000) (Fig. 5e). Moreover, the mRNA levels of Arg-1 (0.78 ± 0.19 fold change vs. 1.46 ± 0.23 fold change, *p* = 0.000), Fizz-1 (1.48 ± 0.32 fold change vs.14.23 ± 0.73 fold change, *p* = 0.000), Ym-1 (1.24 ± 0.44 fold change vs. 2.08 ± 0.72 fold change, *p* = 0.048), and IL-10 (undetected vs. 4.63 ± 2.00 fold change, *p* = 0.001) (Fig. 5d), accompanied with the expression of CD206 (60.77 ± 5.60% vs. 81.33 ± 5.41%, *p* = 0.003) (Fig. 5f), were blocked after LY294002 stimulation.

**Fig. 6:**
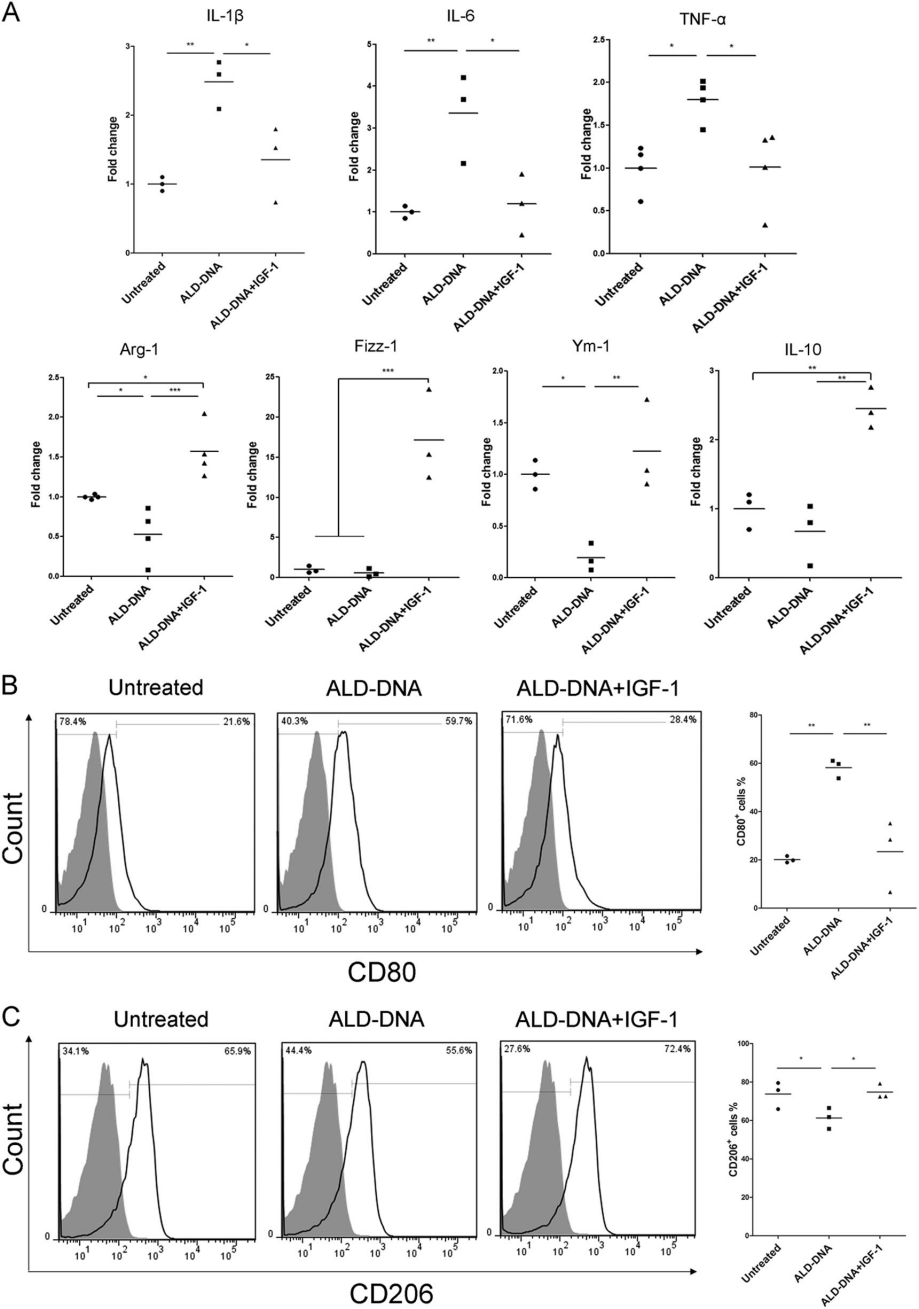
IGF-1 improved the M2 phenotype in imitated SLE macrophages. **a** IGF-1 depressed the mRNA levels of IL-1β, IL-6, and TNF-α, meanwhile, promoted Arg-1, Fizz-1, Ym-1, and IL-10 expression. The frequencies of CD80^+^ cells (**b**) or CD206^+^ cells (**c**) are shown in each histogram. Solid black line indicated CD80 or CD206 staining, and filled grey histogram showed negative controls. *N* = 3–4. *p < 0.05, **p < 0.01, ***p < 0.001

To further confirm the role of PI3K/Akt signaling pathway in M2 activation, we substituted IGF-1 (the specific agonist of Akt) to azithromycin. With the Akt phosphorylation at 30 min and 12 h (Fig. 5c), imitated SLE macrophages treated with IGF-1 only showed reduced mRNA levels of IL-1β (1.35 ± 0.55 fold change vs. 2.48 ± 0.35 fold change, *p* = 0.011), IL-6 (1.19 ± 0.73 fold change vs. 3.35 ± 1.07 fold change, *p* = 0.012) and TNF-α (1.01 ± 0.47 fold change vs. 1.80 ± 0.25 fold change, *p* = 0.011) (Fig. 6a), as well as CD80 expression (23.39 ± 14.86% vs. 58.17 ± 3.84%, *p* = 0.003) (Fig. 6b). At the same time, the mRNA levels of Arg-1 (1.57 ± 0.34 fold change vs. 0.53 ± 0.33 fold change, *p* = 0.000), Fizz-1 (17.14 ± 5.70 fold change vs. 0.58 ± 0.51 fold change, *p* = 0.000), Ym-1 (1.23 ± 0.44 fold change vs. 0.19 ± 0.13 fold change, *p* = 0.004), and IL-10 (2.45 ± 0.29 fold change vs. 0.67 ± 0.45 fold change, *p* = 0.001) (Fig. 6a), accompanied with the expression of CD206 (74.73 ± 3.87% vs. 61.30 ± 5.47%, *p* = 0.026) (Fig. 6c), were up-regulated after IGF-1 treatment. Thus, both signaling data and biological activity experiments indicated that the PI3K/Akt signaling pathway plays an important role in azithromycin-mediated M2 activation in imitated SLE macrophages.

## Discussion

Compared with healthy individuals, SLE patients showed impaired phagocytosis activity in macrophages^17^. The delayed apoptotic cell clearance resulted in secondary necrosis and released massive nucleic acid, which was then be presented to T and B cells, then triggered over activation of immune cells, and ultimately destroyed the organs^3^. According to the above process, we suggest the macrophage dysfunction might be the initiator of the onset of SLE. Thus, we focused on the macrophages in SLE.

It is well known that macrophage derived from mononuclear phagocytic system^29,30^. As the precursor cells, monocytes in the blood migrate to tissues and become macrophages, especially during inflammation^31^. Since it is really difficult to acquire enough macrophages from human organs, we isolated monocytes form peripheral blood instead. Similar to the SLE macrophages^17^, we found the phagocytic activity was extremely decreased in SLE monocytes. Then we compared the phenotype of monocytes between SLE patients and healthy donors. Although the M1 and M2 phenotypes were designed to define macrophages, the specific markers were conclusively expressed on monocytes^32^. Our results showed lower expression of CD14^+^CD163^+^ (M2-like) in SLE monocytes, which is consistent with previous research, however, the previous study reported no difference in the expression of CD86 between patients and healthy donors^32^, but we found extremely higher expression of CD86 in the patients’ monocytes. We supposed the reason might be the patient inclusion criteria, to avoid drug effect as much as possible, our patients were all diagnosed for the first time, only 5 of them had accepted prednisolone treatment before and the therapy duration was strictly controlled within 7 days, however, in their study, most of the patients had accepted prednisolone, hydroxychloroquine, or immunosuppressive therapy, the multiple drugs may affect the final phenotypes. Additionally, the M1 markers (IL-1β, IL-6, TNF-α) significantly increased or showed a higher trend in the patients in our study, while the M2 markers (Arg-1, Fizz-1, IL-10) exhibited a decreased trend. All these data indicated the monocytes from SLE patients expressed a polarized M1 profile. Therefore, we reported the imbalance of M1 and M2 came from the very beginning as the monocytes in SLE patients, the M1-like monocytes were then recruited to tissues and differentiated into M1 macrophages, the redundant M1 “destroyers” resulted in persistent inflammatory which plays a crucial role in the development of SLE.

Depending on our results, we suggested monocytes/macrophages education might be a novel therapeutic approach for SLE. In fact, growing evidence suggested that M2 recovery is beneficial to relieve SLE. For example, the TACI (transmembrane activator and calcium modulator and cyclophilin ligand interactor) deficient MRL-Fas^Lpr^ mice showed a delayed onset of SLE, and adoptively transferred TACI deficient macrophages alleviated lupus nephritis in wild type MRL-Fas^Lpr^ mice, all because the TACI deficient macrophages were M2 dominant^14^. Adoptive transplantation of M2 macrophages could also alleviate SLE severity^16^. However, most of these studies used adoptive transplantation of exogenous cells, thus the curative effect is not sustainable and needs to be constantly replenished. Since the monocytes/macrophages phenotype is dynamic and plastic, we hope to find an approach to regulate the patients’ own monocytes/macrophages.

In the present study, we used azithromycin to regulate M2 phenotype. In 2008, Murphy reported that azithromycin could induce M2 activation for the first time^19^. In the following decade, azithromycin showed magical therapeutic effects in cystic fibrosis, COPD, spinal cord injury, and ischemic stroke injury by promoting M2 differentiation^21,23–25^. However, the role of azithromycin in SLE is still uncertain. Therefore, we detected the hypothesis that azithromycin could act as an immunomodulator in SLE. Considering that the tissue concentration of azithromycin is pretty higher than that of plasma^33,34^, we used SLE macrophages rather than SLE monocytes. The patients’ monocytes were differentiated into macrophages in the presence of M-CSF in vitro, and then stimulated with azithromycin for 48 h. We found for the first time that azithromycin shifted SLE macrophages towards a M2 phenotype, characterized by decreased IL-1β, IL-6, and TNF-α, increased Arg-1, Fizz-1, IL-10, and higher phagocytic activity. Of note, these changes not only indicate the phenotype alteration, they are functional. The improved phagocytic activity might delay the onset of SLE at the very beginning, and the reduced pro-inflammatory factors might help protect against the persistent inflammatory. As for IL-10, it has powerful anti-inflammatory properties and its up-regulation is vital to the limitation of tissue injury by the immune system in response to autoimmunity^35^. Therefore, it is reasonable that azithromycin might relieve SLE by regulating macrophages towards M2 phenotype. This deduction requires further investigation.

To determine the mechanism of azithromycin in regulating macrophages, we used ALD-DNA to imitate SLE in vitro. The ALD-DNA was used for SLE induction by Qiao for the first time^36^. Subsequently, numerous studies related to SLE were carried out based on ALD-DNA induction^10,37,38^. In our study, ALD-DNA-induced macrophages acquired a polarized M1 phenotype which was in agreement with the macrophages from SLE patients, and we found azithromycin exhibited the same function in ALD-DNA-induced macrophages in vitro. Our works showed significant Akt phosphorylation at 30 min and 12 h after azithromycin treatment, suggested Akt activation might be involved. Then we blocked PI3K activation with LY294002 upstream of Akt to inhibited Akt phosphorylation, and found azithromycin-stimulated M2 activation was inhibited afterward, therefore demonstrating that activation of Akt is a key mediator of M2 activation. To further confirm, IGF-1, the specific agonist of Akt, was used as a substitute for azithromycin, and the results demonstrated the key role of Akt activation again. These results coincide with that of others under different inductive conditions^26,28^.

With regard to long-term macrolide therapy, the perturbation about resistant strains of bacteria is inevitable. Fortunately, the therapeutic effect of azithromycin in macrophage modulation has been demonstrated in COPD and cystic fibrosis patients, and no adverse clinical consequences due to bacterial resistance were observed^21,22^. In another cystic fibrosis research, the investigators used 500 mg azithromycin three times per week for an average of 1 year, although the percentage of the bacteria resistant to azithromycin increased, there were no data that displayed the resistance patterns caused any clinical impact^39^. In our study, we only used azithromycin for a short time in vitro, so the studies about bacteriology have not been carried out. We wish to further investigate the role of azithromycin and the possible resistant strains of bacteria in SLE in vivo in the future. And we emphasize that macrophages are not crisply divided into M1 and M2 populations, but rather a continuous spectrum, and even the same population plays diverse roles in different stage. The M2 might be helpful to limit tissue injury in the inflammatory phase, but promote fibrosis in later stage^40^. Therefore, the key point of azithromycin therapy in SLE is to keep dynamic balance of monocytes/macrophages rather than modulate the phenotypes simplex.

In conclusion, this study provides momentous new insights into the biological basis of monocytes/macrophages dysfunction in SLE. Our findings of improved phagocytic activity and reduced inflammation after azithromycin treatment indicate a novel approach to alleviate SLE and provide a rationale to further investigate macrolides as a therapeutic strategy for SLE. Targeting the PI3K/Akt signaling pathway with specific agonist may present a new therapy for manipulating macrophage phenotype in SLE.

## Materials and methods

### Patients and healthy controls

A total of 15 SLE patients participated in this study and all patients satisfied the SLE diagnostic criteria of the American College of Rheumatology (ACR). All patients were diagnosed with SLE for the first time without prednisone and immunosuppressive therapy before or with short-term prednisone therapy (<7 days). Fifteen age- and gender-matched healthy individuals were recruited as healthy controls. Peripheral blood samples were collected from the patients and the healthy donors after obtaining informed consent. Disease activity at the time of sample collecting was assessed by the Systemic Lupus Erythematosus Disease Activity Index (SLEDAI) score. Individuals with other diseases were excluded. Further characteristics of the patients are summarized in Table 1. This project was approved by the Independent Ethics Committee of Huashan Hospital.

**Table 1:** Demographic, clinical, and immunological characteristics of the patients

Characteristics	SLE patients
Age^a^ (years)	33 ± 15 (16–55)
Gender (F/M)	14:1
SLEDAI^a^	5 ± 4 (0–14)
24 h urinary protein^a^ (mg)	1855 ± 4103 (0–12450)
Serum creatinine^a^ (μmol/L)	56.19 ± 26.26 (23.6–130)
Serum urea nitrogen^a^ (mmol/L)	6.55 ± 4.80 (2.3–17.6)
Serum C3^a^ (g/L)	0.78 ± 0.26 (0.45–1.23)
Serum C4^a^ (g/L)	0.14 ± 0.11 (undetected–0.41)
Anti-ANA Ab (%positive)	87
Anti-dsDNA Ab (%positive)	53
Anti-Sm Ab (%positive)	33
WBC^a^ (10^9^/L)	6.65 ± 3.69 (2.58–14.35)
NEU^a^ (%)	70.32 ± 15.91 (37.90–88.20)
Monocytes^a^ (%)	6.43 ± 2.19 (3.60–11.80)
RBC^a^ (10^12^/L)	3.77 ± 0.98 (1.65–4.74)
Hb^a^ (g/L)	111 ± 25 (51–137)
PLT^a^ (10^9^/L)	200 ± 97 (43–417)
Prednisone therapy duration^a^ (days)	1.14 ± 1.99 (0–6)

*SLE* systemic lupus erythematosus, *SLEDAI* Systemic Lupus Erythematosus Disease Activity Index, *Ab* antibody, *WBC* white blood cells, *NEU* neutrophil granulocyte, *RBC* red blood cells, *Hb* hemoglobin, *PLT* platelet

^a^Presented by mean ± standard deviation (SD)

### Sample processing

Peripheral blood mononuclear cells (PBMCs) were isolated from SLE patients and healthy donors with Ficoll-Paque PLUS gradient (GE Healthcare Life Sciences, Pittsburgh, PA, USA). Monocytes from the PBMCs were purified by magnetic separation using CD14 positive selection according to the manufacturer’s instructions (Miltenyi, Cologne, Germany). The purity of CD14^+^ cells was >99% as assessed by flow cytometer. CD14^+^ monocytes were then cultured in Roswell Park Memorial Institute (RPMI) 1640 (Thermo Fisher Scientific, Waltham, MD, USA) supplemented with 10% fetal bovine serum (FBS) (Thermo Fisher Scientific) and 100 ng/ml M-CSF (PeproTech, Rocky Hill, NJ, USA) for 7 days to differentiate into macrophages. Then, a tested concentration of azithromycin (100 μM) (Sigma-Aldrich, St. Louis, MO, USA) was used for another 48 h after careful titration experiments assessing the cell viability. At last, the macrophages were harvested for further detection.

### Cell viability

Human macrophages and Raw 264.7 cells were treated with varying concentrations (10–200 μM) of azithromycin to assess the cell viability. Cell viability was measured by Cell Counting Kit-8 (CCK8) (Dojindo Molecular Technologies Inc., Shanghai, China) assay according to the manufacturer’s instruction. The viability of human macrophages was 88% at 48 h (Fig. S3) and that of Raw 264.7 cells was 82% (Fig. S4). In addition, we detected the mRNA levels of IL-1β, IL-6, TNF-α, and Fizz-1 to assess the efficacy of azithromycin at different concentrations, and the results showed the concentration of 100 μM exhibited the maximum impact (data not shown).

### Phagocytic assay

For the detection of phagocytic activity, macrophages were co-cultured with carboxylate-modified YF FluorSpheres (Thermo Fisher Scientific) at 1:100 dilutions for 2 h. After washing, the uptake of carboxylate-modified YG FluorSpheres was determined by flow cytometer.

### DNA preparation

ALD-DNA was prepared from splenocytes of female BALB/c mice. Briefly, spleens were surgically resected from 6–8-week-old female BALB/c mice, after lapping into single cell suspension, concanavalin A (Con A, Sigma-Aldrich) was added at 5 μg/ml for 6 days to induce apoptosis. Genomic DNAs of the apoptosis cells were extracted with the phenol chloroform method. DNA concentration was determined by absorbance at 260 nm, and the final A260/A280 between 1.8 and 2.0 was approved.

### Apoptosis analysis

Apoptosis of Con A-treated cells was assayed by flow cytometer using the FITC Annexin V Apoptosis Detection Kit (BD Bioscience, Franklin Lake, NJ, USA) according to the manufacturer’s instruction. DNA extracted from Con A-treated cells was examined by horizontal gel electrophoresis.

### Cell culture

Raw 264.7 cells were maintained in Dulbecco’s Modified Eagle Medium (DMEM) (Thermo Fisher Scientific) supplemented with 10% FBS in a 5% CO_2_ incubator at 37 °C. To imitate the SLE environment, cells were incubated with ALD-DNA for 48 h. Azithromycin was added for the whole 48 h incubation period, LY294002 and IGF-1 were added 1 h prior to ALD-DNA. At the indicate time, cells were lysed in cell lysis buffer (CST, Boston, MA, USA) or TRizol reagent (Thermo Fisher Scientific) for following detection.

### Flow cytometry analysis

Membrane expressions of CD86, CD163, CD80, and CD206 were measured in human monocytes and Raw 264.7 cells after being washed in buffer containing 0.1% bovine serum albumin (BSA). Monocytes were gated on the basis of forward and side light scatter and by using a fluorescein isothiocyanate (FITC)-conjugated anti-human CD14 antibody (BD Bioscience) and Fixable Viability Dye (Thermo Fisher Scientific). The following antibodies were used for staining: allophycocyanin (APC)-conjugated anti-human CD86 (BD Bioscience), peridinin chlorophyll protein/cyanin 5.5 (PerCP/Cy5.5)-conjugated anti-human CD163 (BD Bioscience), phycoerythrin (PE)-conjugated anti-mouse CD80 (BD Bioscience), and APC-conjugated anti-mouse CD206 (Thermo Fisher Scientific). Cells were stained in the dark at 4 °C for 30 min. All flow cytometry data were acquired on a BD FACScanto flow cytometer (BD Bioscience) and analyzed by FlowJo software (TreeStar, Inc., Ashland, OR, USA).

### Quantitative real-time polymerase chain reaction (PCR)

Total RNA was extracted from cultured cells with TRIzol reagent according to the manufacturer’s instruction. The cDNA was synthesized using the PrimeScript RT reagent kit (Takara, Otsu, Japan). The expressions of the genes encoding IL-1β, IL-6, TNF-α, Arg-1, Fizz-1, Ym-1, and IL-10 were quantified by real-time PCR using the ABI 7500 system. All gene expression levels were calculated as 2^−ΔΔCt^ using β-actin as the housekeeping gene. Primary primer sequences used in this study are shown in Table 2.

**Table 2:** Primer sequences for quantitative real-time PCR

Gene	Sense primer	Antisense primer
Human β-actin	AGTTGCGTTACACCCTTTCTTG	GCTGTCACCTTCACCGTTCC
Human IL-1β	CATTGCTCAAGTGTCTGAAGCA	CTGGAAGGAGCACTTCATCTGTT
Human IL-6	GGATTCAATGAGGAGACTTGCC	ACAGCTCTGGCTTGTTCCTCAC
Human IL-10	GACTTTAAGGGTTACCTGGGTTG	TCACATGCGCCTTGATGTCTG
Human TNF-α	GAGGCCAAGCCCTGGTATG	CGGGCCGATTGATCTCAGC
Human Arg-1	TGGACAGACTAGGAATTGGCA	CCAGTCCGTCAACATCAAAACT
Human Fizz-1	CCGTCCTCTTGCCTCCTTC	CTTTTGACACTAGCACACGAGA
Mouse β-actin	GGCTGTATTCCCCTCCATCG	CCAGTTGGTAACAATGCCATGT
Mouse IL-1β	GAAATGCCACCTTTTGACAGTG	TGGATGCTCTCATCAGGACAG
Mouse IL-6	ACAACCACGGCCTTCCCTACTT	CACGATTTCCCAGAGAACATGTG
Mouse IL-10	CCAAGCCTTATCGGAAATGA	TTCACAGGGGAGAAATCG
Mouse TNF-α	AAGCCTGTAGCCCACGTCGTA	GGCACCACTAGTTGGTTGTCTTTG
Mouse Arg-1	CAAGACAGGGCTCCTTTCAG	GTAGTCAGTCCCTGGCTTATGG
Mouse Fizz-1	CCTGCTGGGATGACTGCTACT	AGATCCACAGGCAAAGCCAC
Mouse Ym-1	AGAAGGGAGTTTCAAACCTGGT	GTCTTGCTCATGTGTGTAAGTCA

### Western blotting

The expression of Akt (CST), p-Akt (CST) was examined by western blotting. Cells were lysed in 100 μL cell lysis buffer containing phosphatase inhibitors (Roche, Basel, Switzerland) and protease inhibitors (CST) for 10 min on ice. 20 μg of total protein per sample was subjected to 10% SDS-polyacrylamide gels and transferred onto a polyvinylidene fluoride (PVDF) membrane (Millipore, Billerica, MA). After blocking, the membranes were incubated with the primary antibodies at 4 °C overnight, followed by specific HRP-conjugated secondary antibodies for 1 h at room temperature (RT), and finally detected by the enhanced chemiluminescence system (ECL) detection kit (Millipore). Positive immunoreactive bands were normalized by β-actin (CST).

### Statistical analysis

All numeric data were presented as mean ± standard deviation. Significance of differences between multiple groups was assessed by one-way ANOVA, with a LSD multiple comparison using SPSS20.0 software (SPSS Inc., Chicago, IL, USA). Statistical analyses between two groups were performed by Student’s *t* test, and paired *t* test was used for pairing comparison. Values of *p* < 0.05 were considered statistically significant.

## Electronic supplementary material

Supplementary information
