# Supplementary material for: Azithromycin promotes alternatively activated macrophage phenotype in systematic lupus erythematosus via PI3K/Akt signaling pathway
Source: Cell Death Dis. 2018 Oct 22;9(11):1080. doi: 10.1038/s41419-018-1097-5 (PMC6197274; doi:10.1038/s41419-018-1097-5)
Supplement: Supplementary file 1 — Supplementary information [file 41419_2018_1097_MOESM1_ESM.pdf]

The supplementary information showed the validation of ALD-DNA, M2 activation induced by ALD-DNA, and cell viability during azithromycin treatment.

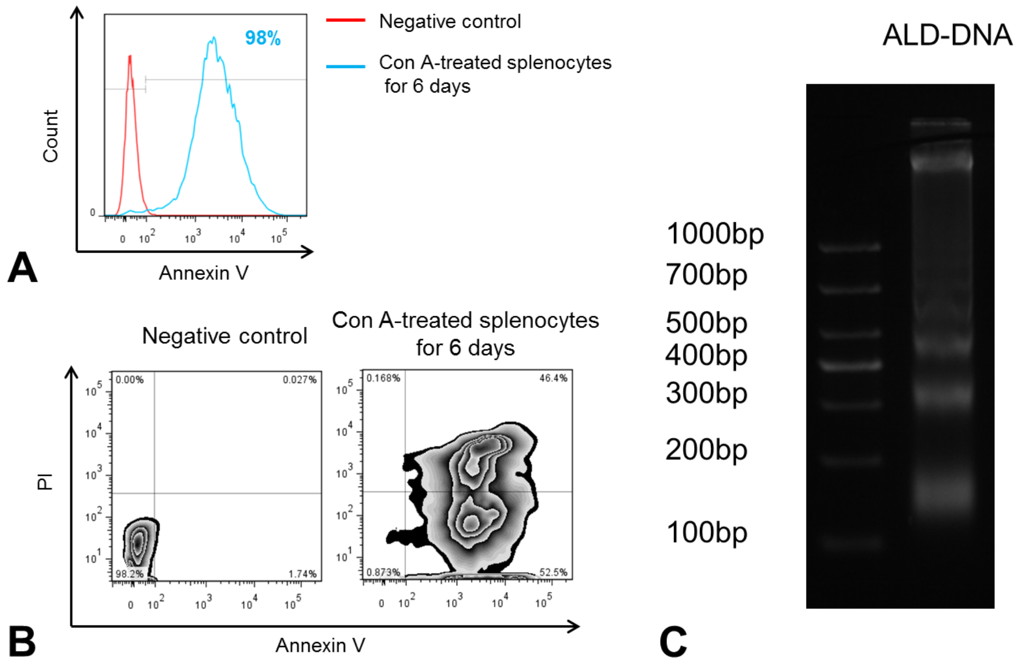

Fig. S1 Validation of ALD-DNA. Approximately 98% splenocytes showed aptosis after stimulation of Con A (A), including about 50% of early apotosis and more than 40% of late apotosis (B). And the extrated DNA exhibited a typical “apotosis ladder” in the gel (C). The red line represents the negative contol, while the blue line represents Con A-treated cells.

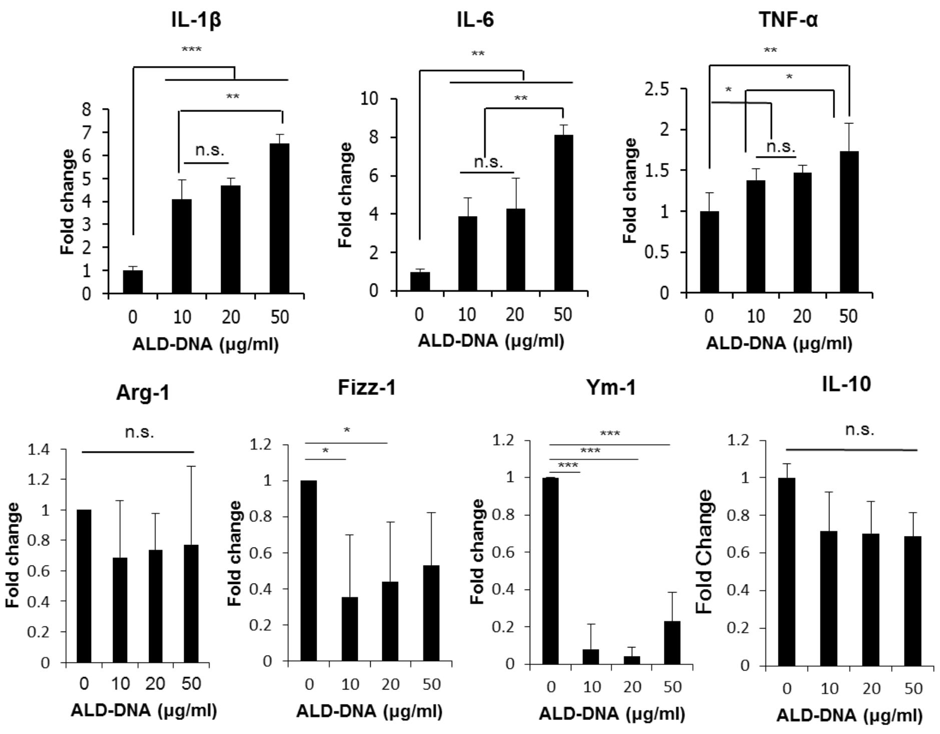

Fig. S2 ALD-DNA induced M1 phenotype in vitro. The mRNA levels of IL-1β, IL-6 and TNF-α were significantly higher in ALD-DNA induced cells from the concentration of 10μg/ml. There were no differences between the concentration of 10μg/ml and 20μg/ml, but at 50μg/ml, it became higher. As the M2 markers, the mRNA levels of Arg-1, Fizz-1, Ym-1 and IL-10 exhibited a decreased trend after ALD-DNA stimulation.*$p < 0.05$, ** $p < 0.01$, *** $p < 0.001$, n.s. not significant.

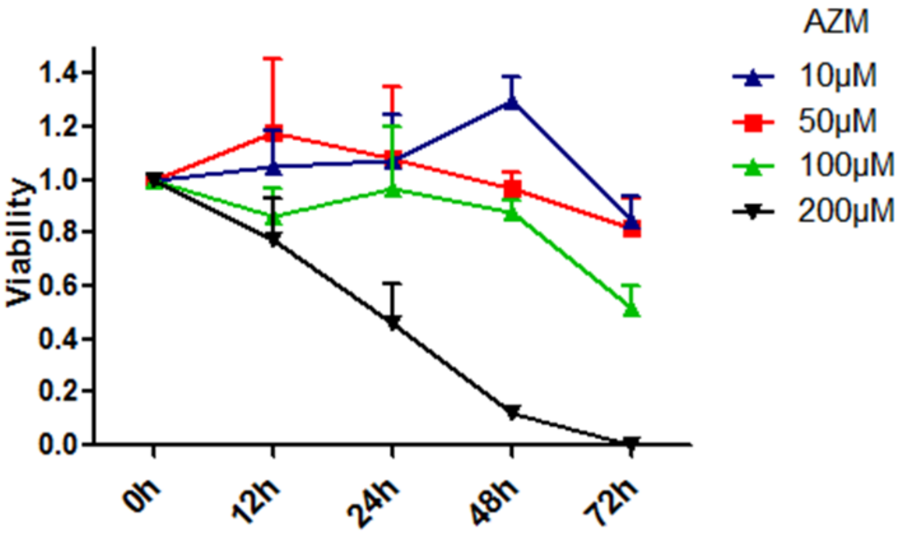

Fig. S3 Human macrophages were treated with varying concentrations (10-200μM) of azithromycin for up to 72 h to assess the cell viability.

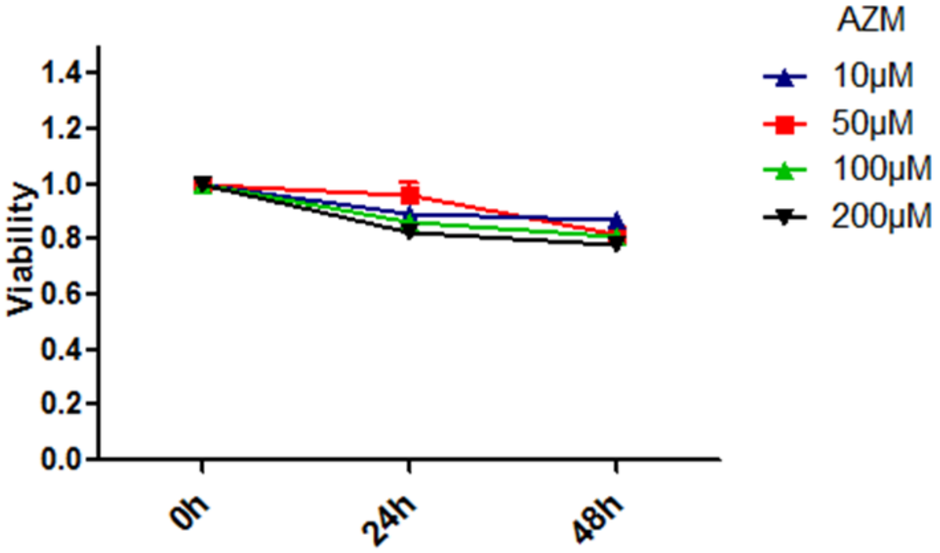

Fig. S4 ALD-DNA-induced Raw 264.7 cells were treated with varying concentrations (10-200μM) of azithromycin for up to 48 h to assess the cell viability.
